# Olfactory Senses Modulate Food Consumption and Physiology in *Drosophila melanogaster*

**DOI:** 10.3389/fnbeh.2022.788633

**Published:** 2022-04-01

**Authors:** Jianzheng He, Wenjuan Tuo, Xueyan Zhang, Yuting Dai, Ming Fang, Ting Zhou, Minghui Xiu, Yongqi Liu

**Affiliations:** ^1^Provincial-Level Key Laboratory for Molecular Medicine of Major Diseases and the Prevention and Treatment with Traditional Chinese Medicine Research in Gansu Colleges and University, Gansu University of Chinese Medicine, Lanzhou, China; ^2^College of Basic Medicine, Gansu University of Chinese Medicine, Lanzhou, China; ^3^Key Laboratory for Transfer of Dunhuang Medicine at the Provincial and Ministerial Level, Gansu University of Traditional Chinese Medicine, Lanzhou, China; ^4^College of Public Health, Gansu University of Chinese Medicine, Lanzhou, China

**Keywords:** olfaction, food consumption, physiology, stress resistance, *Drosophila melanogaster*

## Abstract

Both sensory and metabolic processes guide food intake. Olfactory inputs help coordinate food appreciation and selection, but their role in food consumption and post-feeding physiology remains poorly understood. In this study, using *Drosophila melanogaster* as a model system, we investigated the effects of olfactory sensory neurons (OSNs) on food consumption, metabolism, and stress responses. We found that dysfunction of OSNs affects diverse processes, including decreased food consumption, increased triacylglycerol level, enhanced stress resistance to starvation or desiccation, and decreased cold resistance. Decreased neuropeptide F receptor (NPFR) level or increased insulin activity in OSNs inhibited food consumption, while impaired NPF signaling or insulin signaling in OSNs increased resistance to starvation and desiccation. These studies provide insights into the function of the olfactory system in control of feeding behaviors and physiology.

## Introduction

The regulation of energy homeostasis depends on a dynamic balance between food consumption and energy expenditure (Casanova et al., 2019). This dynamic balance requires the coordinated integration of the peripheral sensory system inputs with internal response systems. The sensory system directs animals toward food sources, guides preferences and satiety experience after consumption, and promotes dietary learning (McCrickerd and Forde, 2016). Animals must weigh both aversive and attractive sensory inputs to evaluate potential food sources. The sensory system undergoes dramatic functional modifications when animals enter different internal states, such as hunger or fullness (Ko et al., 2015). It is important to reveal the mechanisms by which sensory systems, such as the olfactory system and influence feeding.

Olfaction is an ancient sensory system present in animals ranging from *Drosophila* to man. Odorant receptors (ORs) expressed in olfactory sensory neurons (OSNs) can detect and distinguish different olfactory cues (Touhara and Vosshall, 2009). Some OSNs initiate an attractive response to odors, while others mediate the aversive response to odors (Semmelhack and Wang, 2009). Sensitive and accurate detection of food-related odors *via* the olfactory system can guide animals to sources of energy and nutrition. Food-related odors stimulate appetite responses in animals. For instance, the rating of hunger is increased following exposure to food-related odors (Rogers and Hill, 1989). Food-related odors significantly stimulate salivation, insulin release, and gastric acid secretion (Yeomans, 2006). Food odorants also drive animals to consume more food (Reisenman and Scott, 2019). However, the role of the specific OSNs in food intake remains unclear.

The perceptions of olfactory stimuli and behavioral decisions are influenced by internal states. Fasted animals have greater olfactory sensitivity and more active olfactory-driven behaviors than satiated ones (Albrecht et al., 2009; Soria-Gomez et al., 2014), which is due to the decreased secretion of anorexigenic molecules (e.g., insulin, cholecystokinin, and leptin) and increased secretion of orexigenic molecules (e.g., neuropeptide, orexins, and ghrelin) (Soria-Gomez et al., 2014; Peng et al., 2019). Starvation leads to increased behavioral attraction to appetite odors and blunts behavioral avoidance to averse odors (Root et al., 2011; Bracker et al., 2013). The initiation and cessation of feeding behaviors are associated with changes in olfactory sensitivity. Olfactory sensitivity is decreased after the ingestion of food, which is regulated by the satiety signal (Savigner et al., 2009). Insulin receptors are expressed in a subset of OSNs in *Drosophila* flies and vertebrates (Huang et al., 2019; Slankster et al., 2020). In hungry flies, the lower insulin signaling frees production of the short neuropeptide F receptor (sNPFR) to increase sNPF signaling, enhancing the response to odors (Root et al., 2011). In addition, the olfactory system regulates energy homeostasis. Loss of adult OSNs protects against diet-induced obesity in mice, while ablation of IGF1 signaling in OSNs leads to increased adiposity and insulin resistance (Riera et al., 2017).

To further dissect the interplay between the olfactory system and food intake or metabolism, adult flies were used in these studies. *Drosophila* is an excellent model animal for unraveling odor-associated behaviors and physiology, as well as the signaling pathways that mediate feeding and metabolism (Graham and Pick, 2017; Koyama et al., 2020). Our studies reveal that dysfunctional OSNs significantly reduced food intake, increased TAG level, and increased the resistance to starvation and desiccation. We show that NPFR signaling and dInR signaling in OSNs affect the food intake and the stress response. Overall, these findings suggest that specific sets of odor channels, which utilize NPFR signaling and dInR signaling in a gain control mechanism, regulate food intake and the stress response in flies, thereby affecting metabolism.

## Materials and Methods

### Fly Strains and Maintenance

Flies were reared on standard cornmeal dextrose agar food at 25°C, 65% humidity, and 12/12 light/dark cycle. The following fly stocks were obtained from the Bloomington *Drosophila* stock center (BDSC; IN, United States): *w*^1118^ (#5905), *orco*^1^ (#23129), *Orco*-Gal4 (#23292), *Orco*-Gal4 (#26818), *Or42b*-Gal4 (#9971), *Or85a*-Gal4 (#23133), *UAS*-TNT (#28838), *UAS*-Orco (#23145), *UAS*-InR^CA^ (#8263), *UAS*-InR^DN^ (#8251), *UAS*-NPFR RNAi (#25939), and UAS-mCD8:GFP (#5137). The strains were backcrossed for at least five generations with *w*^1118^ to isogenize the genetic background. Adult female flies (3–5 days old) were collected using light CO_2_ anesthesia and allowed to recover for 2 days before further experimentation.

### Food Consumption

The amount of food eaten by single flies was measured using the Capillary Feeder (CAFE) assay (He et al., 2021). Briefly, eight female flies were distributed into each vial with 1% agar at the bottom. Four 5 μl glass capillaries (BRAND, Germany) were inserted into the vial through the lid. The capillaries were filled with 5% sucrose, 5% yeast extract, or both. For the non-starved condition, the amount of liquid food in the capillaries was measured for 24 h. For the starved condition, flies were starved for 21 h before measuring the amount of liquid food for 3 h. One parallel vial void of flies was used as control to determine the extent of liquid evaporation from the capillaries. The total food intake (μl/fly) was calculated as (food consumption − evaporation loss)/number of flies.

### Stress Resistance Assay

Twenty female flies per vial were used to measure the resistance to starvation and desiccation. Replicates (9–10) were set up for each group. Flies were placed in empty vials with 1% agar for starvation resistance and without 1% agar for desiccation resistance. All vials were incubated under constant conditions (25°C, 65% humidity, and 12/12 light/dark cycle). Dead flies were counted every 6–12 h until all flies had died. For resistance to cold stress, flies were placed in empty vials and subjected to cold treatment at 0°C for 4 h, and then, the number of recovered flies was counted every 2 min in a 22°C room.

### Triacylglycerol Assay

The level of triacylglycerol (TAG) was measured as described previously (Tennessen et al., 2014). Briefly, 5 female adult flies per vial were weighed and frozen at −80°C until assay. After homogenizing in PBS + 1% Triton-X, the samples were heated at 70°C for 10 min and then cooled to room temperature. An aliquot was added to the triglyceride reagent (T2449; Sigma, United States), incubated at 37°C for 60 min, added to the free glycerol reagent (F6428; Sigma, United States), and incubated for 5 min at 37°C. TAG content was quantified using a microplate spectrophotometer at OD540 using a standard curve and blank well.

### Statistical Analysis

The data are expressed as mean ± standard error of the mean (S.E.M). Statistical analysis was performed with Prism 7.03 (GraphPad Software, United States). The differences between two groups or among the three groups were examined using Student’s *t*-test or one-way ANOVA followed by Fisher’s protected least significant difference (LSD) test. Survivorships among groups were compared and tested for significance with a Log-rank (Mantel-Cox) test. Differences among different groups were considered to be statistically significant at *p < 0.05, ^**^p < 0.01, and ^***^p < 0.001.

## Results

### Olfactory Sensory Neurons Are Necessary to Regulate Food Intake in Non-starved Flies

**FIGURE 1:**
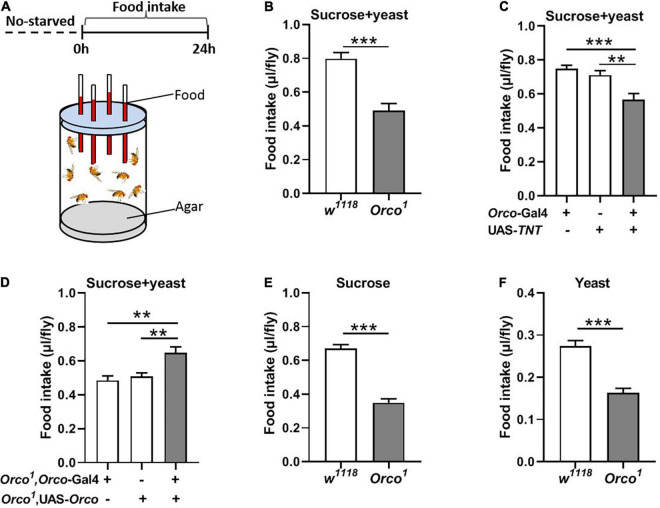
Blocking OSNs decreases the food consumption in non-starved flies. **(A)** Schematic diagram of the CAFE assay for examining food intake in non-starved flies. **(B)** *Orco*^1^ mutant flies consumed less sucrose and yeast extract food than control *w*^1118^ flies (*n* = 15). **(C)** Food intake was decreased after blocking OSNs by expressing TNT in *Orco*-GAL4 driver (*n* = 21). **(D)** Expression of *Orco* in the OSNs in *Orco*^1^ mutants rescued food (5% sucrose and 5% yeast extract) intake (*n* = 15). *Orco*^1^ mutant flies consumed less single sucrose food (*n* = 13) **(E)** and yeast extract food (*n* = 17) **(F)**. Data are represented as mean ± S.E.M. One-sample *t*-test and one-way ANOVA followed by Dunnett’s *post hoc* test were used to analyze the difference. **p < 0.01, and ***p < 0.001.

Olfaction is known to play a major role in food choice. Olfactory sensitivity depends on the internal states, in which starvation encourages animals to search for food *via* increasing olfactory sensitivity (Stafford and Welbeck, 2011; Ko et al., 2015). We wondered whether OSNs regulate the amount of food intake in adult flies. The simple CAFE assay was used to measure food consumption in flies where olfactory signaling was modified (Figure 1A). The results show that total food consumption (5% sucrose + 5% yeast extract) was significantly decreased in *Orco*^1^ mutant flies (Figure 1B) in the non-starved state. Odorant co-receptor (Orco) interacts with the odor-specific receptor and boosts the functional expression of odor-specific receptors in OSNs. We used the UAS-Gal4 system to block chemical synaptic transmission in OSNs by expressing tetanus toxin light chain (TNT). Orco-Gal4/UAS-*TNT* adult flies eat significantly less compared to control flies (Figure 1C). These results indicated that blocking OSNs inhibits food consumption. To confirm that the decreased food intake is indeed due to loss of OSNs function in flies, we restored *Orco* function in *Orco*^1^ mutants using the *Orco*-Gal4 driver (Figure 1D). Expression of *Orco* in OSNs restored food intake in *Orco*^1^ mutants. To eliminate the development effect of OSNs, OSNs were acutely activated by expressing TrpA1 at 30°C during testing. Orco-Gal4/UAS-*TrpA1* adult flies consumed remarkably more food compared to control flies at 30°C, while there was no difference between control flies and experimental flies at 30°C (Supplementary Figure 1). This indicates that acute activation of OSNs promotes food intake. To eliminate the influence of yeast extract odors in food, food was replaced with 5% sucrose or 5% yeast extract (Figures 1E,F). *Orco*^1^ mutants also consumed less food than *w*^1118^ flies. Next, nutrient preference was detected. *w*^1118^ flies and *Orco*^1^ mutants displayed a significant preference for food containing 5% sucrose and 5% yeast extract, compared to food with 5% sucrose (Supplementary Figure 2). However, *Orco*^1^ mutants had a similar preference as *w*^1118^ flies, which suggests that alternation of OSNs plays no role in the regulation of nutrient choice. Taken together, these findings indicate that blocking OSNs inhibits the amount of food consumed in a non-starved state.

### Blocking Olfactory Sensory Neurons Decreases Food Intake in Starved Flies

**FIGURE 2:**
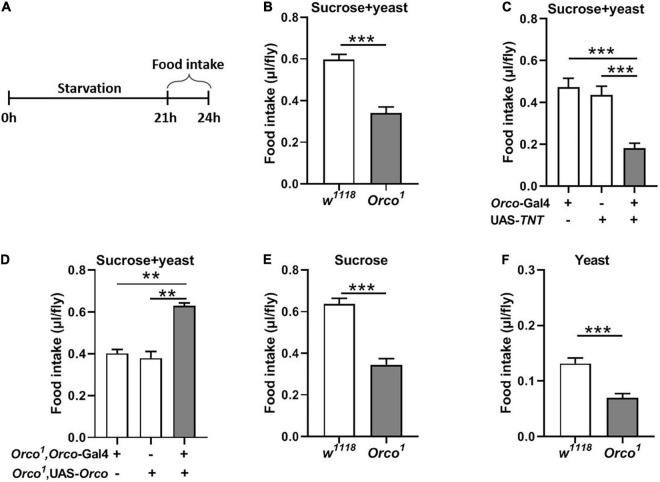
Blocking OSNs decreases the food consumption in starved flies. **(A)** Schematic for food intake of starved flies. **(B)** After 21 h starvation, *Orco*^1^ mutant flies consumed less liquid food containing 5% sucrose and 5% yeast extract than control *w*^1118^ flies (*n* = 12). **(C)** Expression TNT in OSNs decreased food consumption (*n* = 12). **(D)** Expression of *Orco* in OSNs of *Orco*^1^ mutants flies rescued food intake (*n* = 15). Starved *Orco*^1^ mutant flies consumed less single sucrose food (*n* = 19–20) **(E)** or yeast extract food (*n* = 12) **(F)**. **p < 0.01 and ***p < 0.001.

**FIGURE 3:**
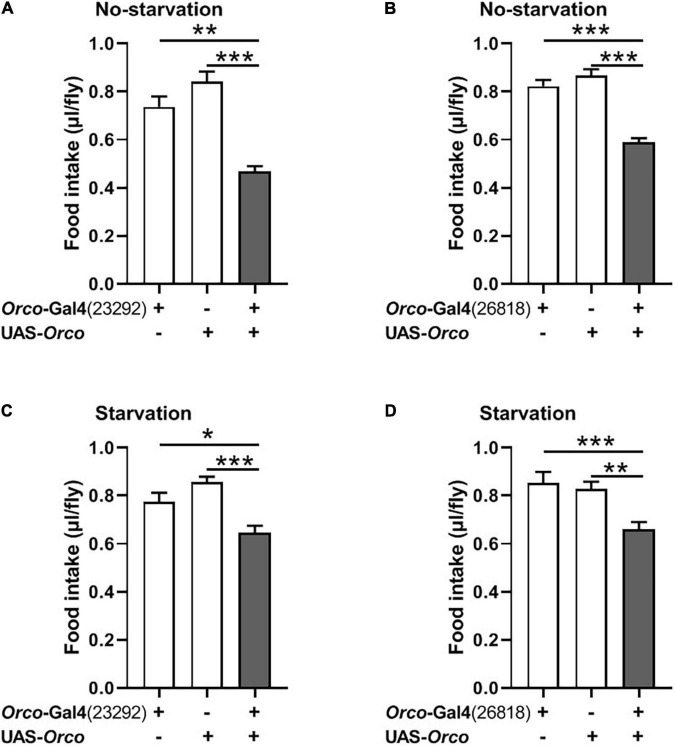
Overexpressing orco in OSNs decreases food consumption. Flies expressing orco in both Orco-GAL4 drive (#23292 and #26818) eaten less food in the non-starved state **(A,B)** and starved state **(C,D)**, *n* = 15–21. One-way ANOVA followed by Dunnett’s *post hoc* test was used to analyze the difference. **p < 0.01 and ***p < 0.001.

To further explore the function of OSNs in food consumption, we measured food intake in starved flies (Figure 2A). After 21 h starvation, *Orco*^1^ mutant flies consumed less food (15% sucrose and 15% yeast extract) than *w*^1118^ flies (Figure 2B, *p* < 0.001). Blocking OSNs by driving the expression of UAS-*TNT* resulted in decreased food consumption compared to corresponding controls (Figure 2C, *p* < 0.01). Restored Orco expression in *Orco*^1^ mutants using the *Orco*-Gal4 driver restored food consumption in *Orco*^1^ mutants (Figure 2D), indicating that the decreased food consumption was due to loss of *orco*-related OSNs function. When the food was replaced by single 5% sucrose or 5% yeast extract, starved *Orco*^1^ mutants still consumed less food than *w*^1118^ control flies (Figures 2E,F). Restored *Orco* expression in *Orco*^1^ mutants using the *Orco*-Gal4 driver restored food consumption in *Orco*^1^ mutants (Figure 3D), indicating that the decreased food consumption was due to loss of orco-related OSNs function. When the food was replaced by single 5% sucrose or 5% yeast extract, starved *Orco*^1^ mutants still consumed less food than *w*^1118^ control flies, indicating that the odor of yeast extract was not the reason for altered food consumption. Thus, these results suggest that blocking OSNs inhibits the amount of food consumed in the starved state.

### Overexpression of Orco in Olfactory Sensory Neurons Inhibits the Food Intake

We next asked whether overexpression of *Orco* in OSNs would promote food consumption in flies. Expression of *Orco* in OSNs using the *Orco*-Gal4 driver (#23292) significantly reduced food intake in non-starved flies (Figure 3A). The same phenotype was found when using another *Orco*-Gal4 driver (#26818) to overexpress *orco* in flies (Figure 3B). In addition, after 21 h of starvation, expressing *Orco* in OSNs remarkably decreased food consumption (Figures 3C,D). Therefore, overexpressing *Orco* in OSNs leads to decreased food consumption.

### Or85a and Or42b OSNs Have No Function in the Regulation of Food Consumption

**FIGURE 4:**
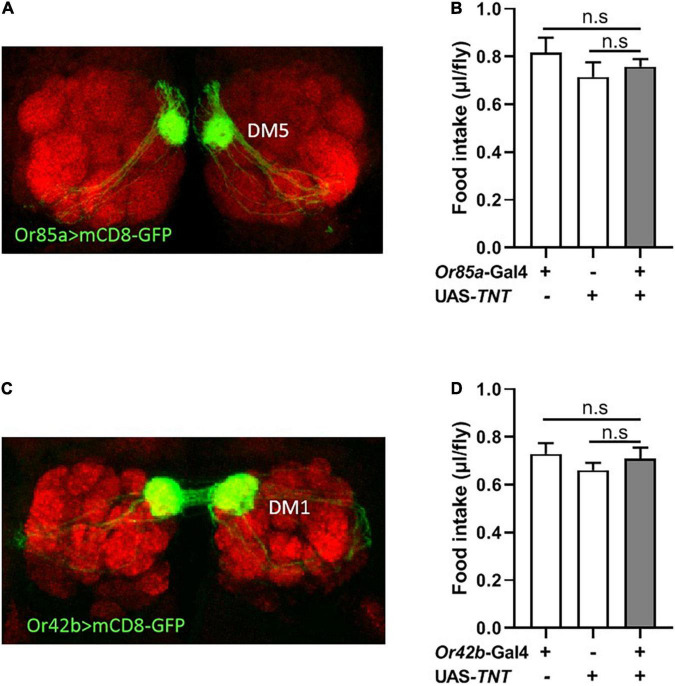
Or85a OSNs regulates food consumption. **(A)** Expression of Or85a OSNs in DM5 of antennal lobe. *Or85a*-GAL4 drives *UAS-mCD8-GFP* expression (Green). **(B)** The food consumption was not affect after expression TNT in Or85a OSNs (*n* = 21). **(C)** Expression of Or42b OSNs in DM1 of antennal lobe. *Or42b*-GAL4 drives *UAS-mCD8-GFP* expression (Green). **(D)** No effect on food intake after expression TNT in Or42b OSNs (*n* = 15). One-way ANOVA followed by Dunnett’s *post-hoc* test were used to analyze the difference.

Previous studies in food search have shown that activation of the DM5 glomerulus, innervated by Or85a OSNs, triggers aversion to odors, while activation of the DM1 glomerulus, innervated by Or42b OSNs, regulates attraction to odors in flies (Semmelhack and Wang, 2009; Ko et al., 2015). Therefore, we next investigated whether modulation of Or85a or Or42b OSN signaling would alter food intake in flies. Visualizing the Gal4 expression domain of the *Or85a*-Gal4 line using a UAS-mCD8:GFP transgene, Or85a OSNs projected into the DM5 glomerulus (Figure 4A). Blocking Or85a OSNs by expressing TNT in flies did not affect the food consumption (Figure 4B). In addition, we found that the DM1 glomerulus was innervated by Or42b OSNs (Figure 4C). When Or42b OSNs were blocked by expressing TNT, the food consumption was not affected in flies (Figure 4D). Therefore, these results suggest that Or85a and Or42b OSNs play no role in the regulation of food intake.

### Insulin and NPF Signaling in Olfactory Sensory Neurons Participate in Food Consumption

**FIGURE 5:**
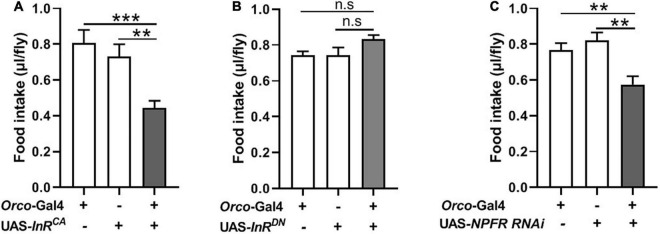
Modulating expression of InR and NPFR in OSNs affect food intake. **(A)** Increasing InR activity in OSNs by driving a UAS-*InR*^CA^ decreased food consumption (*n* = 20). **(B)** No effect on food intake after decreasing InR activity (*n* = 12). **(C)** Decreasing NPFR expression in OSNs inhibited food intake (*n* = 18). One-way ANOVA followed by Dunnett’s *post-hoc* test were used to analyze the difference. **p < 0.01 and ***p < 0.001.

InR expression and NPFR expression have been clearly demonstrated at terminals of adult fly OSNs (Lee et al., 2017; Slankster et al., 2020). Previous studies found that insulin signaling plays important roles during both starvation and olfactory behavior in flies and mammals (Enell et al., 2010; Slankster et al., 2020). NPF enhances the olfactory sensitivity of OSNs and increases appetite behavior in flies (Wang et al., 2013; Lee et al., 2017). We, therefore, investigated the role of insulin and NPF signaling in OSNs on food consumption. We used the UAS-Gal4 system to manipulate InR and NPFR levels in OSNs. InR levels were increased or reduced in OSNs by driving a UAS-*InR*^CA^ or a UAS-*InR*^DN^. Increasing InR activity in OSNs resulted in decreased food consumption compared to corresponding controls (Figure 5A). Decreased InR level did not affect consumption in flies (Figure 5B). Next, NPFR levels were reduced in OSNs by driving a UAS-NPFR-*RNAi.* Reducing NPFR levels in OSNs remarkably decreased food consumption (Figure 5C). These results suggest that insulin and NPF signaling in OSNs have an important role in the regulation of food consumption in flies.

### Abnormal Olfactory Sensory Neurons Signaling Enhances Starvation Resistance

**FIGURE 6:**
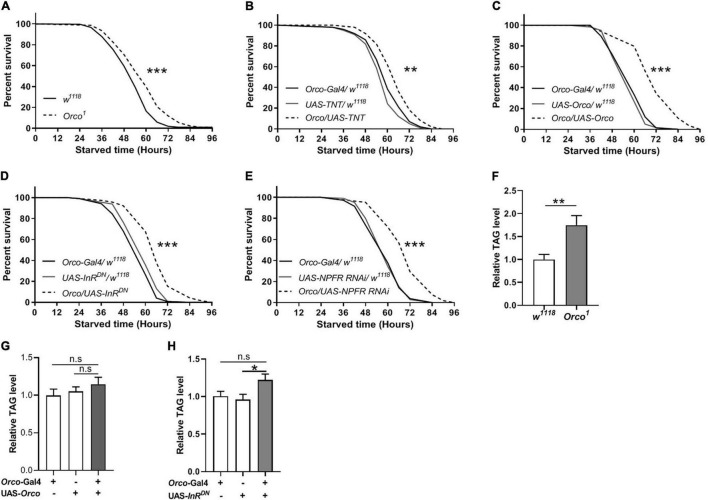
OSNs regulate starvation resistance and lipid metabolism. **(A)** *Orco*^1^ mutant flies had longer survival lifespan under starved condition than *w*^1118^ flies (*n* = 9 vials/group). **(B)** Blocking OSNs by expressing TNT enhanced the starvation resistance (*n* = 9 vials/group). The resistance to starvation was increased after expressing Orco in OSNs **(C)** (*n* = 11–12). Decreasing InR activity **(D)** or decreasing NPFR level **(E)** in OSNs extended the survival lifespan under starved condition (*n* = 10). **(F)** *Orco*^1^ mutant flies had higher triacylglycerol (TAG) level than *w*^1118^ flies (*n* = 9). The level of TAG did not affect after expressing Orco **(G)** and decreasing InR activity **(H)** (*n* = 9–10). *p < 0.05, **p < 0.01, and ***p < 0.001.

Given that manipulation of OSNs signaling can modulate food intake in flies, we next tested whether they also affect starvation resistance and lipid metabolism. *Orco*^1^ mutants exhibited a significant increase in survival rate compared with *w*^1118^ flies (Figure 6A). Blocking OSNs signaling also extended survival time (Figure 6B). Unexpectedly, overexpression of *Orco* in OSNs remarkably increased the survival rate (Figure 6C). Reducing InR levels in OSNs resulted in an increased survival rate compared to corresponding controls (Figure 6D). Decreasing NPFR levels in OSNs also increased the survival rate under starvation conditions (Figure 6E). These results suggest that abnormal OSNs signaling in flies leads to increased resistance to starvation, which is related to insulin and NPF signaling. The observation of enhanced starvation resistance further prompted us to investigate whether body fat deposition was affected by OSNs. TAG levels in *Orco*^1^ mutants were significantly elevated when compared to the *w*^1118^ flies (Figure 6F). Flies in which *orco* was overexpressed in OSNs had similar TAG levels with flies having normal OSNs function (Figure 6G). Reducing InR levels in OSNs slightly increased the TAG levels, but the difference was not significant (Figure 6H). Therefore, altering OSNs signaling or reducing InR levels in OSNs can enhance the resistance to starvation, likely by modulating lipid metabolism.

### Abnormal Olfactory Sensory Neurons Signaling Alters Desiccation and Cold Resistance

**FIGURE 7:**
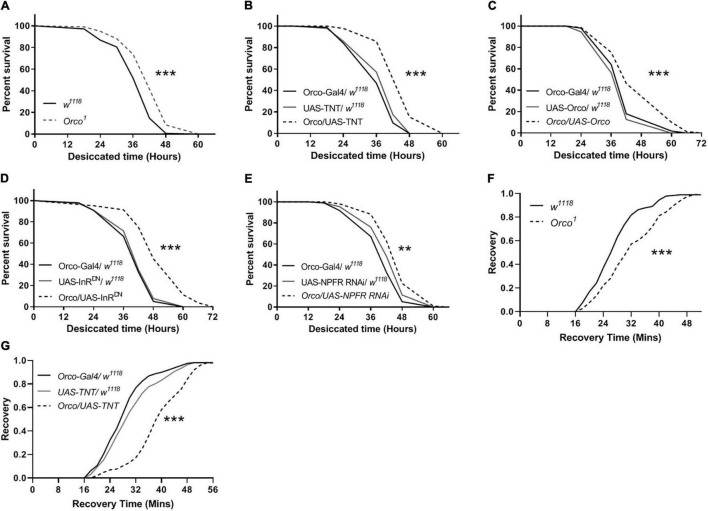
OSNs mediate desiccation and cold resistances. The survival lifespan under desiccated condition was extended in *Orco*^1^ mutant flies **(A)**, after expressing TNT in OSNs **(B)**, after expressing Orco in OSNs **(C)** (*n* = 9–10 vials/group). Decreasing InR activity **(D)** or decreasing NPFR level **(E)** in OSNs enhanced the desiccation resistance (*n* = 8–9). The recovery time after cold stimulation was extended in *Orco*^1^ mutant flies **(F)**, or after expressing TNT in OSNs **(G)** (*n* = 9–12). Log-rank (Mantel-Cox) test were used to determine the significant differences. **p < 0.01 and ***p < 0.001.

To further establish a role for OSNs in the regulation of stress resistance, we investigated whether disorder in olfactory function is sufficient to alter the stress response to desiccation. Compared with *w*^1118^ flies, *Orco*^1^ mutants had a higher survival rate in desiccated conditions (Figure 7A). Flies in which OSNs were blocked lived longer than their parental controls in desiccated conditions (Figure 7B). Expressing Orco in OSNs significantly extended the survival time (Figure 7C). When InR or NPFR signaling was reduced in OSNs, flies showed an increased survival rate (Figures 7D,E). In addition, the stress response to cold was detected in flies. *Orco*^1^ mutants took more time to recover after cold stress as compared to *w*^1118^ flies (Figure 7F). Blocking OSNs by expressing TNT in flies also extended the recovery time after 4 h of cold stress (Figure 7G). Therefore, altering OSNs signaling or reducing InR levels in OSNs enhances the resistance to desiccant, and blocking OSNs decreases the resistance to cold stress.

## Discussion

In this study, we investigated the roles of OSNs in food consumption and stress responses in *Drosophila melanogaster*. We found that defects in OSNs decreased food consumption, increased the TAG level, increased resistance to starvation and desiccation, and decreased cold stress. Or85a and Or42b OSNs played no role in the regulation of food intake. In addition, altering NPFR or insulin signaling in OSNs also modulated food consumption and stress response to starvation or desiccation. Overall, these findings imply a potential relationship between olfactory sensory signaling and animal physiology.

Hunger increases olfactory performance to facilitate the retrieval and ingestion of food in animals (Soria-Gomez et al., 2014). Our results show that flies with loss of functional OSNs consumed less food containing sucrose and yeast extract than control flies and were consistent with the results in which blocking OSNs decreased the consumption of single sucrose food or yeast extract food. This suggests that the odor of yeast extract did not affect the food intake regulated by OSNs. In addition, restored OSNs function in *Orco*^1^ mutants rescued the decreased food intake. Previous studies indicated that food-related odors act as one of the complex environmental influences on appetite and lead to short-term overeating in human and *Drosophila* (Soria-Gomez et al., 2014). The rating of hunger is increased following exposure to food-related odors (Rogers and Hill, 1989). Recent studies found that loss of olfactory input dramatically increases taste sensitivity, which is starvation-independent (Junca et al., 2021). Thus, blocking olfactory signaling inhibits food intake, which may be due to inhibiting appetite behavior. It also may be possible that when OSNs are blocked in flies, the information of food-related odors may not be transferred to the center region of the brain that regulates feeding, resulting in decreased food consumption. Surprisingly, overexpression of *Orco* in all OSNs that express the olfactory co-receptor Orco decreased food consumption. This unexpected result raises the possibility that some OSNs that regulate aversive response are activated and may override the function of other OSNs that mediate the attractive response; second, the increase of OSNs signaling would lead to sensory-specific satiety. Previous studies have shown that prolonged sensory exposure to foods contributes to meal termination and is associated with lower food intake in *Drosophila* and mammals (de Graaf and Kok, 2010; Lushchak et al., 2015); finally, overexpression of *Orco* in OSNs may lead to dysfunction of OSNs, resulting in decreased food consumption. When we activated OSNs by expressing *TrpA1* at 30°C, the amount of food was remarkably increased. These results suggest that overexpression of *Orco* in OSNs does not activate OSNs, but results in dysfunction of OSNs. In starved flies, the sensitivity is increased in Or42b OSNs and is reduced in Or85a OSNs (Ko et al., 2015). Activation of Or85a OSNs induces an aversive response to odors, while activation of Or42b OSNs triggers attraction to odors (Semmelhack and Wang, 2009). However, we found that blocking Or85a or Or42b OSNs does not affect the amount of food consumed. The specific OSNs that regulate feeding enhancement need to be further determined.

Previous studies found that NPF enhances the olfactory sensitivity of OSNs and promotes appetitive odor-induced feeding in *Drosophila* larvae (Wang et al., 2013; Lee et al., 2017). In this study, decreasing NPF signaling by knocking down NPFR in adult flies leads to a reduction in food intake. This result is in line with several other studies in flies and mammals. Overexpression of NPF in flies and NPY injection in the hypothalamus of rats resulted in increased food consumption (Wahlestedt et al., 1993; Lee et al., 2004). Together, these studies indicate that NPF signaling in OSNs positively regulates foraging and feeding behavior in flies. In addition, lower insulin signaling enhances responses to odors by increasing sNPF signaling in OSNs of starved flies (Wahlestedt et al., 1993; Lee et al., 2004). InR is expressed in a subset of OSNs in *Drosophila* flies and vertebrates (Huang et al., 2019; Slankster et al., 2020). We found that expressing an active dInR in OSNs resulted in decreased food consumption in adult flies, which was consistent with findings with larvae flies (Huang et al., 2019; Slankster et al., 2020). It is reasonable to speculate that NPFR and InR levels in OSNs could contribute to differential modulation of OSNs based on the animal’s internal state, which in turn impacts food consumption. NPF signaling and insulin signaling in OSNs positively or negatively regulate feeding in flies.

To increase body fat, either energy has to increase or energy expenditure has to decrease (Spiegelman and Flier, 2001). The loss function of OSNs significantly decreased food intake and elevated the levels of TAG in flies, which suggests that increased TAG appears to occur mainly by the reduced energy expenditure. We also found that the loss function of OSNs extended the resistance to starvation. Therefore, blocking OSNs signaling leads to decreased lipid metabolism. Previous studies have shown that loss of olfactory function significantly extended the lifespan of *Drosophila* (Libert et al., 2007). Dietary restriction has been shown to extend lifespan in almost all model organisms, including *Drosophila* (Libert et al., 2007). Thus, it is possible that decreased lipid metabolism induced by decreasing OSNs signaling promotes flies to live longer. Interestingly, overexpression of *Orco* in OSNs remarkably extended the resistance to starvation or desiccation and had no effect on regulating TAG levels, suggesting that dysfunction of OSNs mainly decreases energy output. Inactivated insulin signaling remarkably increased resistance to starvation in flies (Broughton et al., 2005). Consistently, we found that reducing InR levels in OSNs resulted in increased lifespan in flies exposed to starvation and slightly increased the TAG level in flies, which indicates that insulin signaling in OSNs regulates lipid metabolism. In *Drosophila*, knockdown of NPF in dorsolateral peptidergic neurons (DLPs) or NPF mutants resulted in increased starvation resistance (Broughton et al., 2005). Knockdown of NPFR in OSNs significantly increased starvation resistance. Additionally, dietary restriction (DR) has been shown to increase stress resistance, which may lead to improved health (Wan et al., 2003; Bross et al., 2005). We also found that abnormal OSNs signaling enhanced the desiccation resistance, indicating that OSNs play roles in the regulation of water homeostasis in flies. Blocking OSNs signaling decreased the resistance to cold stress in flies. These results support the conclusion that the olfactory system serves a critical function as a detector for the external environment and as an early warning system to trigger defense responses that are essential for survival (Koike et al., 2021). Therefore, the olfactory system helps flies to sense and adapt to the external environment.

In conclusion, our study demonstrates that stable olfactory signaling input is necessary to regulate feeding behavior and is related to lipid metabolism. NPF signaling and insulin signaling in OSNs have an important role in maintaining the internal state. These studies can help us better understand how animals adapt to their external environment by modulating olfactory sensory input.

## Data Availability Statement

The original contributions presented in the study are included in the article/Supplementary Material, further inquiries can be directed to the corresponding author/s.

## Author Contributions

JH, YL, and MX conceived and designed the study. WT, JH, MF, TZ, XZ, and YD performed the experiments and analyzed the data. JH and MX wrote the manuscript. YL reviewed and edited the manuscript. All authors contributed to the article and approved the submitted version.

## Conflict of Interest

The authors declare that the research was conducted in the absence of any commercial or financial relationships that could be construed as a potential conflict of interest.

## Publisher’s Note

All claims expressed in this article are solely those of the authors and do not necessarily represent those of their affiliated organizations, or those of the publisher, the editors and the reviewers. Any product that may be evaluated in this article, or claim that may be made by its manufacturer, is not guaranteed or endorsed by the publisher.

## Supplementary Material

Supplementary Figure 1 Activating OSNs promote the food consumption in non-starved flies. Food intake was increased when activating OSNs by expressing *TrpA1* in *Orco*-GAL4 driver at 30°C (*n* = 12–15).

Supplementary Figure 2 Blocking OSNs has no function in nutrient preference. *Orco*^1^ mutant flies and *w*^1118^ flies all prefer 5% sucrose with 5% yeast extract diet than 5% sucrose diet.
