# Supplementary figures and images for: Olfactory Senses Modulate Food Consumption and Physiology in Drosophila melanogaster
Source: Front Behav Neurosci. 2022 Apr 1;16:788633. doi: 10.3389/fnbeh.2022.788633 (PMC9011337; doi:10.3389/fnbeh.2022.788633)

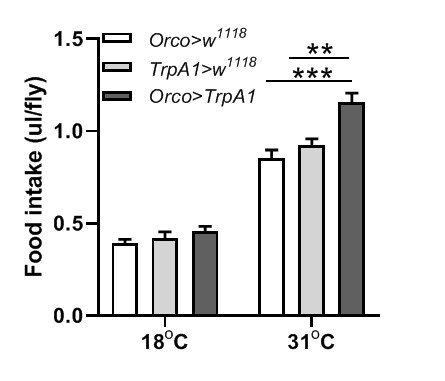

Supplement: Supplementary Figure 1 — Activating OSNs promote the food consumption in non-starved flies. Food intake was increased when activating OSNs by expressing TrpA1 in Orco-GAL4 driver at 30°C (n = 12–15). [file Image_1.JPEG]

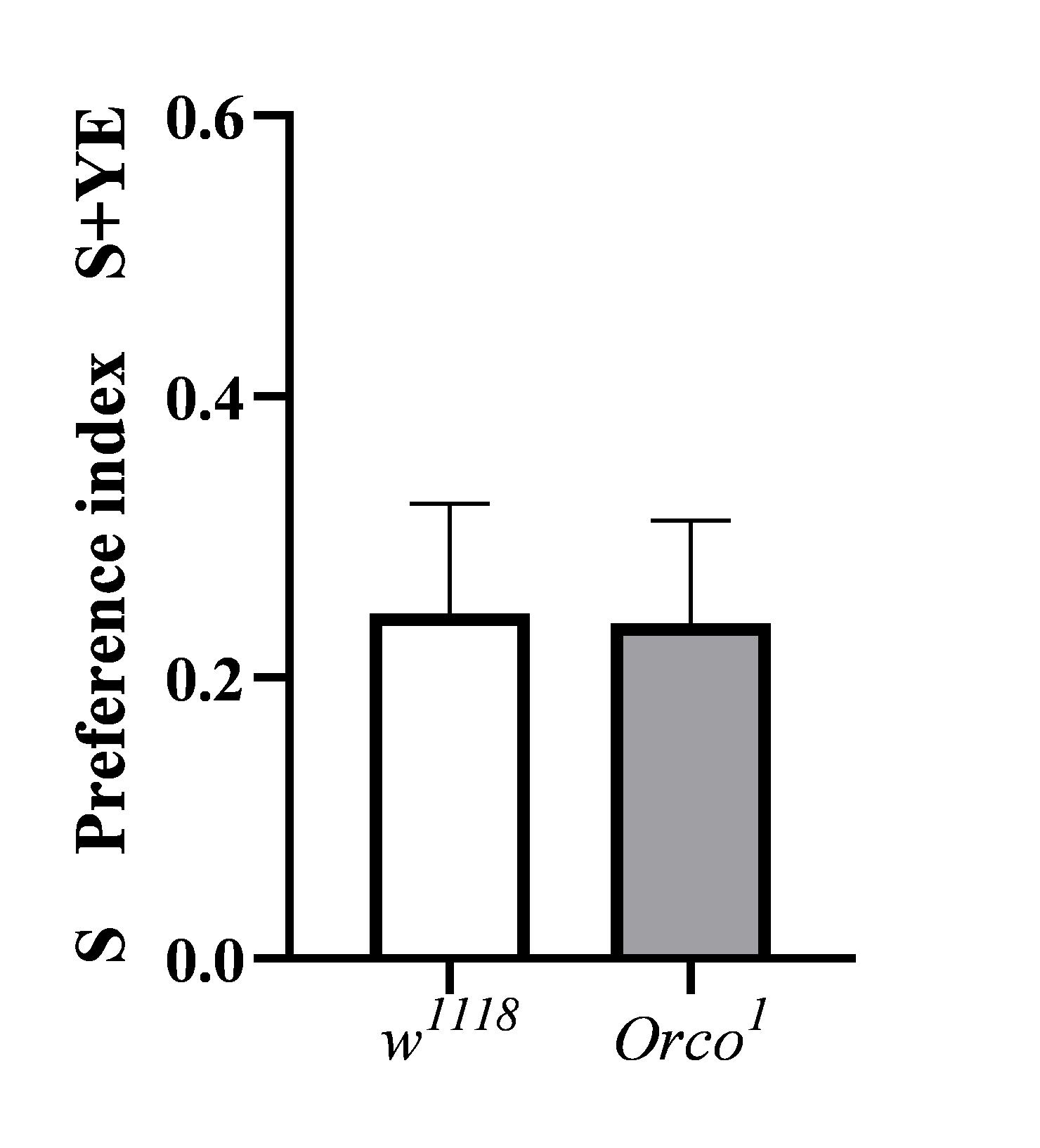

Supplement: Supplementary Figure 2 — Blocking OSNs has no function in nutrient preference. Orco1 mutant flies and w1118 flies all prefer 5% sucrose with 5% yeast extract diet than 5% sucrose diet. [file Image_2.JPEG]
